# PHYSICAL ACTIVITY INTERVENTIONS FOR PEOPLE LIVING WITH DEMENTIA: A SCOPING REVIEW

**DOI:** 10.1093/geroni/igae098.0688

**Published:** 2024-12-31

**Authors:** Sarah McKiddy

**Affiliations:** University of Washington, Seattle, Washington, United States

## Abstract

Dementia poses significant challenges globally, necessitating effective interventions to mitigate its impact on individuals and societies. Physical activity has emerged as a promising avenue for enhancing physical and cognitive function among people living with dementia. This scoping review aimed to evaluate the characteristics and outcomes of various physical activity interventions in this population including articles from Australia, Belgium, Germany, New Zealand, Sweden, the United States, and Japan, reflecting a global effort to address the complexities of considerations for physical activity interventions for dementia. Predominantly utilizing randomized control trials (RCTs), the studies explored interventions ranging from strength and balancing exercises to multicomponent training, targeting individuals aged 50 and older diagnosed with Alzheimer’s disease, other forms of dementia, or mild cognitive impairment. Notably, interventions primarily involved in-person sessions, with a few incorporating remote platforms, emphasizing the importance of accessibility and flexibility in delivering interventions to diverse populations. Results indicated improvements in physical function, cognitive performance, and quality of life, suggesting the potential of physical activity as a multifaceted intervention for dementia management. Despite methodological variations and limitations observed across studies, the findings highlighted the promising role of physical activity in enhancing the well-being of individuals living with dementia. Future research should focus on addressing methodological inconsistencies, expanding diversity in study populations, and exploring optimal intervention strategies to maximize the benefits of physical activity in dementia care.

